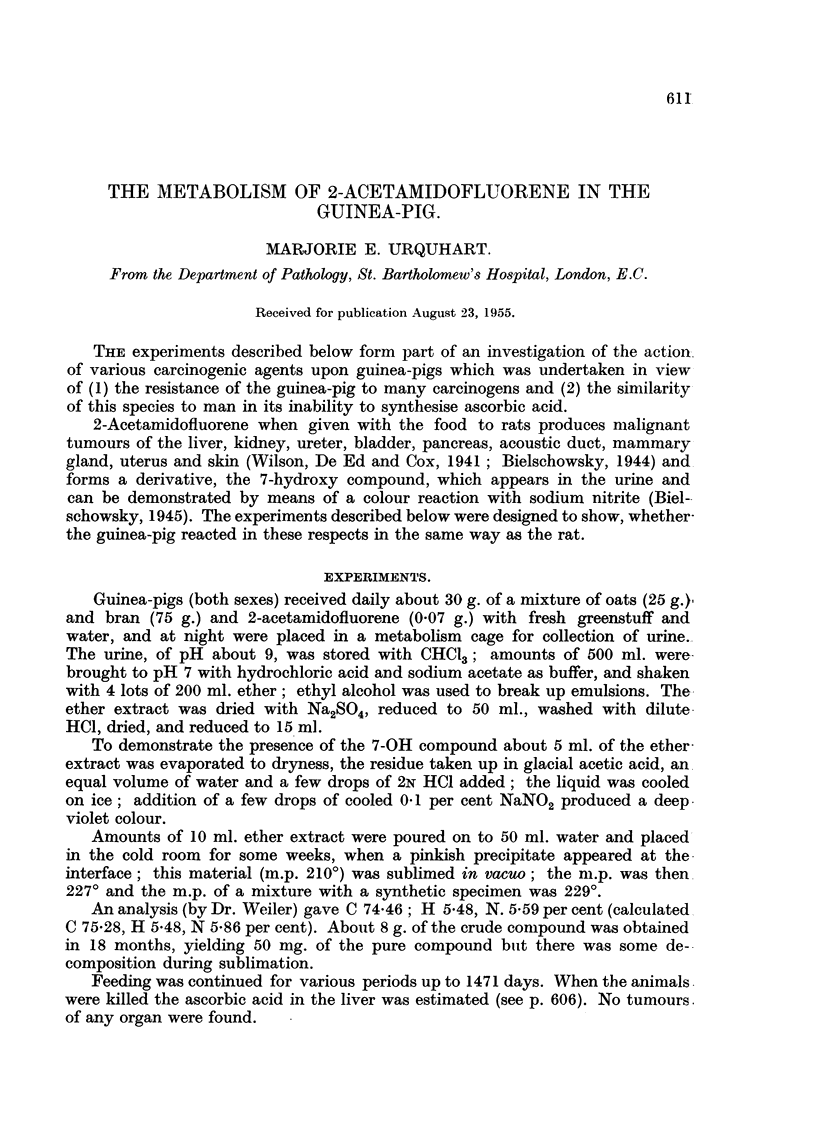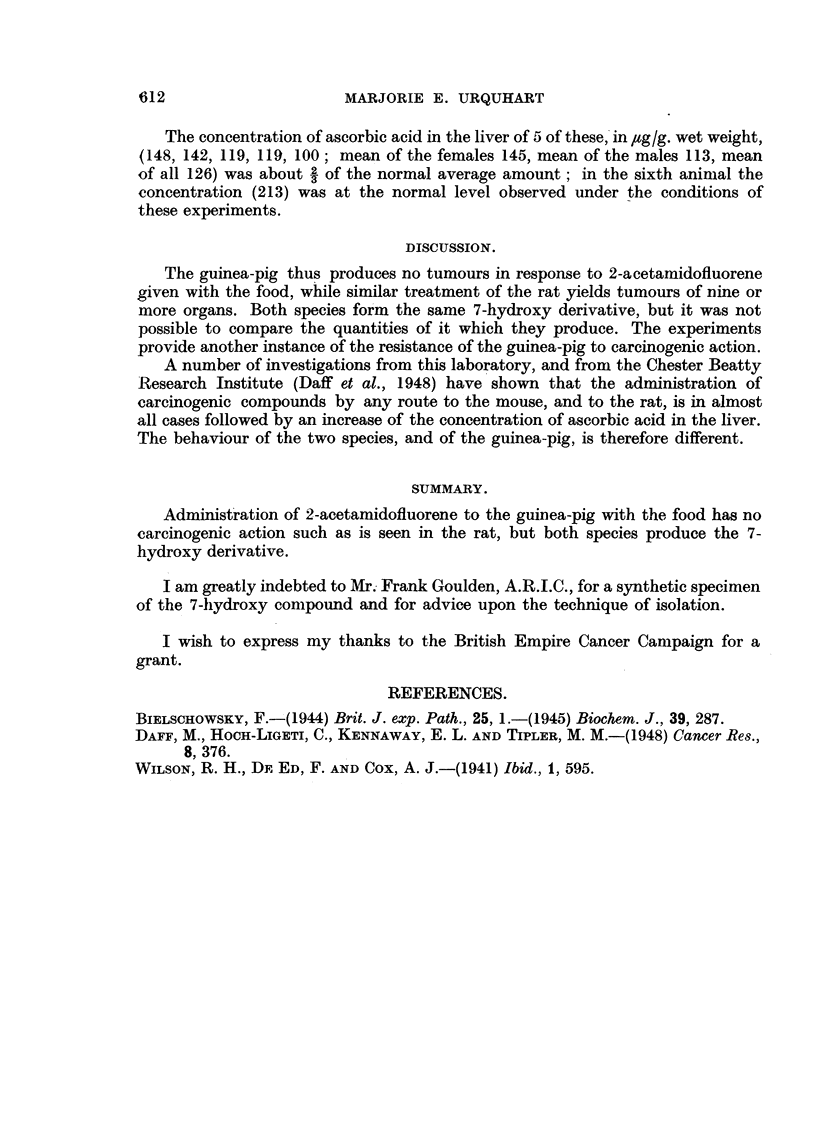# The Metabolism of 2-Acetamidofluorene in the Guinea-Pig

**DOI:** 10.1038/bjc.1955.63

**Published:** 1955-12

**Authors:** Marjorie E. Urquhart


### THE METABOLISM OF 2-ACETAMIDOFLUORENE IN THE GUINEA-PIG.

MARJORIE E. URQUHART.

*From the Department of Pathology, St. Bartholomew's Hospital, London, E.C.*

Received for publication August 23, 1955.

THE experiments described below form part of an investigation of the action of various carcinogenic agents upon guinea-pigs which was undertaken in view of (1) the resistance of the guinea-pig to many carcinogens and (2) the similarity of this species to man in its inability to synthesise ascorbic acid.

2-Acetamidofluorene when given with the food to rats produces malignant tumours of the liver, kidney, ureter, bladder, pancreas, acoustic duct, mammary gland, uterus and skin (Wilson, De Ed and Cox, 1941 ; Bielschowsky, 1944) and forms a derivative, the 7-hydroxy compound, which appears in the urine and can be demonstrated by means of a colour reaction with sodium nitrite (Bielschowsky, 1945). The experiments described below were designed to show, whether the guinea-pig reacted in these respects in the same way as the rat.

### EXPERIMENTS.

Guinea-pigs (both sexes) received daily about 30 g. of a mixture of oats (25 g.) and bran (75 g.) and 2-acetamidofluorene (0·07 g.) with fresh greenstuff and water, and at night were placed in a metabolism cage for collection of urine. The urine, of pH about 9, was stored with $CHCl_3$ ; amounts of 500 ml. were brought to pH 7 with hydrochloric acid and sodium acetate as buffer, and shaken with 4 lots of 200 ml. ether ; ethyl alcohol was used to break up emulsions. The ether extract was dried with $Na_2SO_4$, reduced to 50 ml., washed with dilute HCl, dried, and reduced to 15 ml.

To demonstrate the presence of the 7-OH compound about 5 ml. of the ether extract was evaporated to dryness, the residue taken up in glacial acetic acid, an equal volume of water and a few drops of 2N HCl added ; the liquid was cooled on ice ; addition of a few drops of cooled 0·1 per cent $NaNO_2$ produced a deep violet colour.

Amounts of 10 ml. ether extract were poured on to 50 ml. water and placed in the cold room for some weeks, when a pinkish precipitate appeared at the interface ; this material (m.p. 210°) was sublimed *in vacuo* ; the m.p. was then 227° and the m.p. of a mixture with a synthetic specimen was 229°.

An analysis (by Dr. Weiler) gave C 74·46 ; H 5·48, N. 5·59 per cent (calculated C 75·28, H 5·48, N 5·86 per cent). About 8 g. of the crude compound was obtained in 18 months, yielding 50 mg. of the pure compound but there was some decomposition during sublimation.

Feeding was continued for various periods up to 1471 days. When the animals were killed the ascorbic acid in the liver was estimated (see p. 606). No tumours of any organ were found.

The concentration of ascorbic acid in the liver of 5 of these, in $\mu g/g$. wet weight, (148, 142, 119, 119, 100 ; mean of the females 145, mean of the males 113, mean of all 126) was about $\frac{2}{3}$ of the normal average amount ; in the sixth animal the concentration (213) was at the normal level observed under the conditions of these experiments.

### DISCUSSION.

The guinea-pig thus produces no tumours in response to 2-acetamidofluorene given with the food, while similar treatment of the rat yields tumours of nine or more organs. Both species form the same 7-hydroxy derivative, but it was not possible to compare the quantities of it which they produce. The experiments provide another instance of the resistance of the guinea-pig to carcinogenic action.

A number of investigations from this laboratory, and from the Chester Beatty Research Institute (Daff *et al.*, 1948) have shown that the administration of carcinogenic compounds by any route to the mouse, and to the rat, is in almost all cases followed by an increase of the concentration of ascorbic acid in the liver. The behaviour of the two species, and of the guinea-pig, is therefore different.

### SUMMARY.

Administration of 2-acetamidofluorene to the guinea-pig with the food has no carcinogenic action such as is seen in the rat, but both species produce the 7-hydroxy derivative.

I am greatly indebted to Mr. Frank Goulden, A.R.I.C., for a synthetic specimen of the 7-hydroxy compound and for advice upon the technique of isolation.

I wish to express my thanks to the British Empire Cancer Campaign for a grant.

### REFERENCES.

BIELSCHOWSKY, F.—(1944) *Brit. J. exp. Path.*, **25**, 1.—(1945) *Biochem. J.*, **39**, 287.
DAFF, M., HOCH-LIGETI, C., KENNAWAY, E. L. AND TIPLER, M. M.—(1948) *Cancer Res.*, **8**, 376.
WILSON, R. H., DE ED, F. AND COX, A. J.—(1941) *Ibid.*, **1**, 595.